# Using delayed decoupling to attenuate residual signals in editing filters

**DOI:** 10.5194/mr-2-475-2021

**Published:** 2021-06-21

**Authors:** Kenneth A. Marincin, Indrani Pal, Dominique P. Frueh

**Affiliations:** 1 Department of Biophysics and Biophysical Chemistry, Johns Hopkins School of Medicine, Baltimore, MD 21205, USA; a current address: Department of Biochemistry and Molecular Biophysics, Kansas State University, Manhattan, KS 66506, USA

## Abstract

Isotope filtering methods are instrumental in biomolecular nuclear magnetic resonance (NMR) studies as they isolate signals of chemical moieties of interest within complex molecular assemblies. However, isotope filters suppress undesired signals of isotopically enriched molecules through scalar couplings, and variations in scalar couplings lead to imperfect suppressions, as occurs for aliphatic and aromatic moieties in proteins. Here, we show that signals that have escaped traditional filters can be attenuated with mitigated sensitivity losses for the desired signals of unlabeled moieties. The method uses a shared evolution between the detection and preceding preparation period to establish non-observable antiphase coherences and eliminates them through composite pulse decoupling. We demonstrate the method by isolating signals of an unlabeled post-translational modification tethered to an isotopically enriched protein.

## 1 Introduction

Nuclear magnetic resonance (NMR) has become a mainstay of biomolecular studies, notably because its non-invasive nature makes it particularly suited to study interactions between biomolecules at the atomic level, such as protein–protein (Sprangers and Kay, 2007), protein–DNA/RNA (Kalodimos et al., 2004), and protein–small molecule interactions (Meyer and Peters, 2003). A key to this success is the ability to control the isotopic labeling of the molecules participating in the interactions and use spin dynamics to isolate signals of interest in otherwise crowded spectra. Thus, in mixtures of isotopically labeled ( ${}^{15}N{/}^{13}C$ ) and unlabeled ( ${}^{14}N{/}^{12}C$ ) components, signals from the labeled components can either be selected (isotopic editing) or eliminated (isotopic filtering) (Otting and Wüthrich, 1990). Imperfections in isotopic filtering of labeled signals are recognized as a common challenge, and unsuppressed signals can bias interpretations of results. We have been motivated to overcome this challenge within the framework of our studies of nonribosomal peptide synthetases (NRPSs), a family of enzymatic systems that produce important pharmaceuticals such as antibiotics (bacitracin, vancomycin), anticancer agents (epothilones), or immunosuppressants (cyclosporine) (Finking and Marahiel, 2004; Süssmuth and Mainz, 2017).

**Figure 1:**
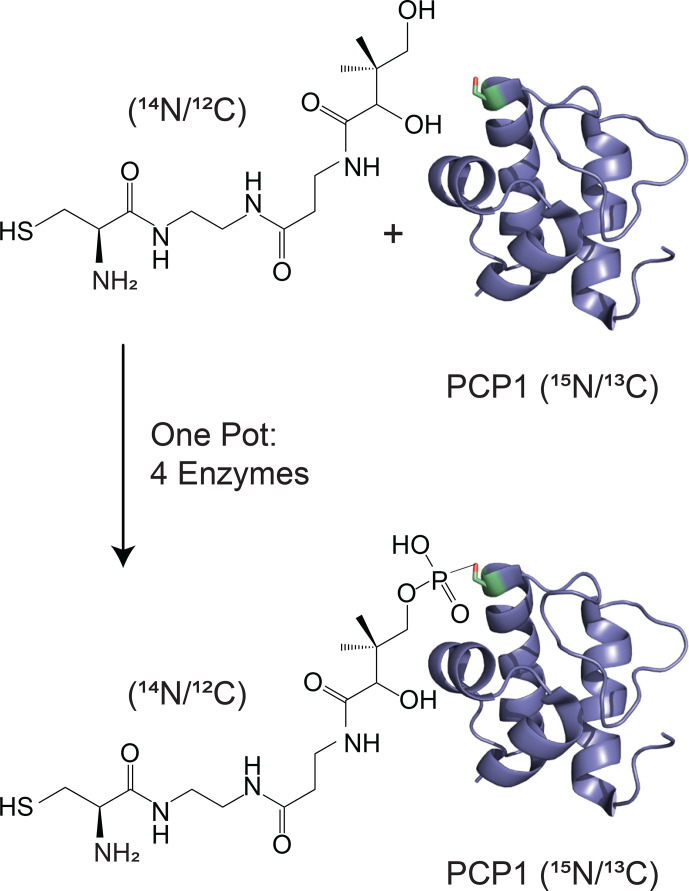
Isotopic labeling scheme used in our studies. Isotopically enriched PCP1 ( ${}^{15}N{/}^{13}C$ ) is chemoenzymatically tethered with an unlabeled pantetheine analogue ( ${}^{14}N{/}^{12}C$ ) harboring a cysteine substrate ( ${}^{14}N{/}^{12}C$ ). See Sect. 3.2 for more details. PCP1 solution structure: PDB 5U3H (Harden and Frueh, 2017). Analogue structures were made in ChemDraw (PerkinElmer Informatics).

Isotopic filtering will enable the study of interactions between NRPSs and their substrates. NRPSs employ domains called carrier proteins (CPs) to covalently tether substrates and shuttle them between partner catalytic domains. These domains are organized in contiguous modules, and substrates attached to CPs on sequential modules are condensed such that an upstream CP donates its substrate to a downstream CP, which then harbors an extended intermediate. The process is iterated until product formation. In this, carrier proteins are covalently modified with a 20 Å long phosphopantetheine (PP) group (Lambalot et al., 1996) which gets covalently loaded with an amino acid substrate. We and others have found that some CPs interact transiently with their tethered substrates (Goodrich et al., 2015; Jaremko et al., 2015) such that the phosphopantetheine group and its attached substrate sample both an undocked state and a docked state. The finding is significant as the modular architecture of NRPSs is suitable for producing new pharmaceuticals by engineering NRPSs to incorporate new substrates, and these transient interactions may need to be preserved in artificial NRPSs. In the current work, we focus on the 9.6 kDa carrier protein (PCP1) isolated from yersiniabactin synthetase, which natively harbors a cysteine substrate (Gehring et al., 1998). In order to study interactions between PCP1 and the phosphopantetheine group and its attached cysteine substrate, we have chemoenzymatically (Kittilä and Cryle, 2018; Worthington and Burkart, 2006) attached the unlabeled PP group and substrate to PCP1 enriched in ${}^{15}N$ and ${}^{13}C$ isotopes (Fig. 1). The NMR linewidths of the tethered moiety indicate that the arm does not tumble independently from the protein core but is also not rigidly docked onto the protein, in line with a transient interaction. Unfortunately, we found that traditional filtering methods leave undesired labeled protein signals in the spectra of the PP arm and its substrate, confusing data interpretation and hampering future mechanistic studies. Our immediate objective is to attenuate these residual signals and mitigate sensitivity losses for the targeted signals of unlabeled moieties, which will be particularly important for future studies of PCP1 engaging with its larger partner domains.

Isotopic filtering is a tested tool for detecting interactions between labeled and unlabeled molecules but is inefficient in presence of large variations in scalar couplings. Many methodologies have been implemented to filter signals from labeled molecules in direct or indirect dimensions, reviewed in Breeze (2000) and Robertson et al. (2009). As our immediate objective is to obtain 1-D proton spectra of unlabeled moieties, we do not consider methods that exploit evolutions in indirect dimensions, and here we focus solely on filters for the detected dimension. The existing methods that achieve our aim rely on the same common principle: single-quantum coherences of protons coupled to heteronuclei can evolve into antiphase coherences, while those of protons in unlabeled molecules remain in-phase. The filtering pulse sequences are designed to preserve the latter and eliminate the former. This filtering methodology targets a specific range of scalar couplings and is limited when very different scalar couplings need to be targeted, as occurs for proton–carbon scalar couplings that vary from 120 to 220 Hz in proteins and 140 to 220 Hz in nucleic acids (Zwahlen et al., 1997). Three alternative solutions can overcome this challenge. Low-pass J filters (Kogler et al., 1983) can be incorporated (Ikura and Bax, 1992), elements targeting different couplings can be applied sequentially (Breeze, 2000; Gemmecker et al., 1992; Zangger et al., 2003), and the correlation between chemical shift and scalar couplings can be exploited through adiabatic pulses (Eichmüller et al., 2001; Kupče and Freeman, 1997; Valentine et al., 2007; Zwahlen et al., 1997). In all cases, further suppression of labeled molecule signals comes at the cost of sensitivity losses, which we attempted to mitigate.

Here, we present a method to attenuate undesired signals in the detected dimension that escaped traditional filters with minimal increase in the length of the pulse sequence. The method relies on allowing evolutions under scalar couplings during both the detection period and existing adjacent preparation periods such as water-suppression schemes. We show that, although only applicable to a narrow range of scalar couplings, this strategy satisfyingly removes spurious signals of the labeled protein core of PCP1 and provides reliable spectra of its tethered unlabeled phosphopantetheine group and substrate. As an immediate application, we implemented our modification in a 2-D total correlation spectroscopy (TOCSY) experiment to remove misleading correlations. Due to the simplicity of its implementation, we predict that our method will readily improve studies of unlabeled moieties attached covalently or non-covalently to larger, labeled biomolecules.

## 2 Theory

### Using delayed decoupling to suppress residual signals of protons coupled to heteronuclei

Our objective is to attenuate residual signals from coupled spins that have escaped filters with minimal or no increase in the lengths of pulse sequences. In our immediate application, we repurpose a water-suppression scheme in addition to the detection period to function as an additional filter. In this section, we discuss how signals are attenuated by sharing evolution under scalar couplings between these two periods and applying composite pulse decoupling once undesired coherence become antiphase.

Delayed decoupling can be used to dampen the signals of spins coupled to heteronuclei. Delayed decoupling has previously been used to enhance sensitivity in solution NMR (Rößler et al., 2020), and the partitioning of adjacent undecoupled and decoupled periods has been used to determine carbon hybridization states in solid-state NMR spectra (Alla and Lippmaa, 1976). Decoupling without delay has been used to suppress undesired antiphase coherences for filters immediately preceding detection (Yang et al., 1995). Here, we use delayed decoupling to improve isotope filtering while minimizing sensitivity losses due to relaxation. Although our method is best implemented through experimental optimizations (*vide infra*), its mechanism can be described through simple principles. We first consider a simple pulse-and-acquire experiment, in which decoupling is applied after a time τ=1/|2J| and assume that an initial in-phase single-quantum coherence has become fully antiphase by the end of τ . Further, we only consider the signal of a single multiplet component. In absence of delayed decoupling, the signal is simply

1 $$s(t)=e^{\left(i\omega_{0}-R\right)t},$$

where, for simplicity, $\omega_{0}$ implicitly includes contributions from the scalar coupling, i.e., for a two-spin system on resonance $\omega_{0}=\pm \pi \text{J}$ , and R is a transverse relaxation rate incorporating all relevant relaxation mechanisms. The signal in the frequency domain is that described in every NMR textbook:

2 $$S\left(\omega \right)=\frac{-1}{i\left(\omega_{0}-\omega \right)-R}.$$

Note that we chose to use this compact form rather than the more conventional representation as a sum of real and imaginary components to remind the reader that the intensity at $\omega =\omega_{0}$ does not have any imaginary component, which will be exploited below and prevent unnecessary derivations. Thus, substituting ω with $\omega_{0}$ in Eq. (2) gives

3 $$I\left(\omega =\omega_{0}\right)=\frac{1}{R}.$$

**Figure 2:**
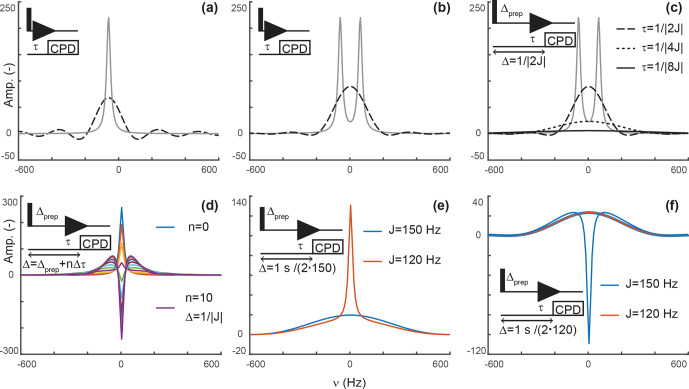
Principles of editing through delayed decoupling. **(a)** Applying decoupling once coherences are antiphase truncates their free induction decay (FID) and attenuates their signals (dashed line), as shown here for the isolated component of a doublet. **(b)** The two components combine into a broadened and attenuated shape (dashed line). The analytical expressions of Eqs. (2) (solid gray line) and (4) (dashed black line) were used in panels **(a, b)**. **(c)** Further attenuation is obtained when evolution into antiphase coherences is shared between a preparation period and detection as shown through simulations. The total evolution, Δ , was set to 1/|2J| , with evolutions during detection τ=1/|2J| (dashed line), 1/|4J| (dotted line), and 1/|8J| (solid line). In panels **(a–c)**, spectra without delayed decoupling are shown in gray for reference. **(d)** Simulation where the duration Δ is arrayed for a fixed preparation period $\Delta_{\mathrm{prep}}=1/|4\text{J}|$ , and τ ranges from zero to 3/|4J| leading to Δ=1/|J| in 10 increments Δτ of 3/|40J| . This simulation predicts the results seen in Fig. 4b. In panels **(a–d)**, J is set to 120 Hz. **(e)** A delayed decoupling targeting 150 Hz leads to residual positive in-phase signals for spins with couplings at 120 Hz. **(f)** A delayed decoupling targeting 120 Hz leads to negative residual in-phase signals for couplings at 150 Hz. In panels **(e, f)**, $\Delta_{\mathrm{prep}}=1/|4\text{J}|$ and τ is set to 1/|4J| for the targeted J, i.e., half of the total duration Δ .

Again, this is a solution that should be familiar to every reader. When decoupling is applied after τ , the signal only evolves during τ , after which decoupling prevents evolution of antiphase coherences into detected in-phase terms. In our scheme, s(t) is zero after τ but has a conventional evolution during τ . This description is reminiscent of discussions of truncation artifacts, which, in the frequency domain, lead to the convolution of Lorentzian signals with a sinc function. That is, signals are now both attenuated and accompanied with sinc wiggles (Fig. 2a).

An analytical expression can be obtained by integrating Eq. (1) between 0 and τ to give

4 $$S_{\tau }(\omega )=\frac{e^{\left(i\omega_{0}-R\right)\tau }-1}{i\left(\omega_{0}-\omega \right)-R},$$

providing the spectrum shown in Fig. 2a. The intensity at $\omega =\omega_{0}$ is

5 $$I_{\tau }(\omega =\omega_{0})=\frac{1-e^{-R\tau }}{R},$$

such that the delayed decoupling attenuates the intensity with

6 $$I_{\tau }(\omega =\omega_{0})/I\left(\omega =\omega_{0}\right)=1-e^{-R\tau }.$$

That is, the efficiency of the attenuation is in part governed by relaxation and in part by the delay before decoupling is applied. For a system of two scalar-coupled spins, the signal corresponds to the sum of two shapes described by Eq. (4) and separated by J, as shown in Fig. 2b. Although, in the example we discussed, τ is set to 1/|2J| , our objective is to implement delayed decoupling in pulse sequences with preparation periods preceding detection. Equation (5) indicates that these preparation periods will enhance attenuations if undesired coherences are allowed to evolve under scalar couplings before detection such that smaller τ values are needed to reach antiphase coherences.

Figure 2c depicts simulations describing how delayed decoupling affects signals of coherences when they also evolve under scalar couplings before detection. Here, we found the analytical solution to be of little use, as the shape of the detected signal is a complex combination of multiplet components that are dephased during the preparation period, and the quality of the attenuation can no longer be assessed through the intensity at $\omega =\omega_{0}$ . Instead, we ran simulations where the total duration from establishing a single-quantum coherence to applying decoupling is kept at $\Delta =\Delta_{\mathrm{prep}}+\tau =1/|2\text{J}|$ , where $\Delta_{\mathrm{prep}}$ is the evolution period preceding detection and τ is the delay described above, i.e., separating the beginning of signal detection from the application of decoupling. In Fig. 2c, τ takes the values 1/|2J| (when $\Delta_{\mathrm{prep}}$ is zero), 1/|4J| , and 1/|8J| . In agreement with Eq. (5), Fig. 2c indicates that signals are most attenuated when evolution towards antiphase occurs predominantly during the preparation period. However, it also shows that the shapes of the signals change dramatically, with a line broadening accompanied with attenuation of sinc wiggle artifacts.

Figure 2d shows a simulation where the duration between establishing the coherence and applying decoupling, Δ , is arrayed for a target scalar coupling (here, 120 Hz). Figure 2c reports exclusively on sharing the evolution under scalar couplings between the preparation and detection periods but does not report on incomplete conversion into antiphase operators, as would occur when a variety of scalar couplings must be considered or when arraying delays to optimize signal attenuations, as performed experimentally. Figure 2d shows that when decoupling is applied before reaching 1/|2J| , a residual positive in-phase signal is detected, whereas a negative in-phase signal emerges past 1/|2J| . These signals are combined with the shapes described above resulting from truncated antiphase evolution, leading to unconventional line shapes, in particular, when Δ exceeds the optimal value of 1/|2J| . Note that, because of this behavior, zero amplitude is achieved on resonance for delays Δ slightly exceeding 1/|2J| , when a residual negative in-phase signal compensates for the truncated antiphase signal (as seen for the signal in green in Fig. 2d). Indeed, experimentally, we found that the value of τ selected through visual inspection typically exceeds 1/|2J| as signal suppression appears more efficient at those values than at 1/|2J| . Similarly, delayed decoupling filters tuned for a target scalar coupling will distort the shapes of signals with off-target couplings. This drawback is illustrated in Fig. 2e and f for filters targeting either the maximal aliphatic scalar coupling for ${}^{1}H{-}^{13}C$ in proteins (around 150 Hz, on average) or the minimal value (120 Hz for methyls), respectively. As a corollary, the quality of the filter's bandwidth includes an aesthetic component which cannot reasonably be quantified. This aspect will be illustrated experimentally below, with the major conclusion being that the filter is solely to be used to supplement existing filters and not replace them.

In conclusion to this section, delayed decoupling can dramatically reduce the signals of spins coupled to heteronuclei, but off-target couplings will display undesirable line shapes. Thus, although not robust as a stand-alone technique, the method is ideal to supplement existing isotopic filters where it can target residual unwanted signals at reduced costs in sensitivity for the signals of unlabeled moieties.

## 3 Methods

### 3.1 Simulations

Simulations were coded in Matlab (MathWorks Inc., 2018). Briefly, the evolution of the density operator is calculated in a single-transition and polarization operator basis, only accounting for the detected terms ${\text{LS}}^{\alpha }$ and ${\text{LS}}^{\beta }$ . Propagation is achieved through the following matrix:

$$M=\left(\begin{array}{cc} i\omega_{0\alpha }+R & 0\\ 0 & i\omega_{0\beta }+R \end{array}\right).$$

For simplicity, the simulation is only performed on resonance such that $\omega_{0\alpha }=\pi \text{J}$ and $\omega_{0\beta }=-\pi \text{J}$ . R is a transverse relaxation rate, either obtained through calculations or estimated experimentally. In our simulations, we used a value of 2π⋅14 Hz estimated from the linewidth of an isolated signal. The density operator at the time of detection was calculated through $\mathrm{exp}(-M\Delta_{\mathrm{prep}})S_{0}$ , where $S_{0}$ was set to (1;1) . With the simplifications described above, this propagation describes the evolution into antiphase operators with concomitant refocusing of chemical shift evolutions. An array recording the evolution during the detection period is then calculated through exp⁡(-Mdt)S(t-dt) , first with free evolution, and from the time t=τ , with intermittent exchange of ${\text{LS}}^{\alpha }$ and ${\text{LS}}^{\beta }$ , thus simulating decoupling through a perfect 180 ${}^{\circ }$ pulse. The relationship between the dwell time and dt is set by the number of inversions during the dwell time, and in the present work the dwell is 2dt for one inversion per dwell. Departure from the simulations includes cross-correlated autorelaxation and autocorrelated cross-relaxation effects that would differentiate the relaxation rates of each component and mix the two components, respectively. Further differences with experimental implementations include evolutions during composite pulse decoupling (CPD) sequences and off-resonance effects.

### 3.2 Sample preparation

#### 3.2.1 PCP1 cloning, isotope labeling, expression, and purification

The PCP1 construct used in this study was prepared as described in Harden and Frueh (2017) utilizing the 1402–1482 gene fragment from the *Yersinia pestis* irp2 gene (accession number AAM85957). Briefly, PCP1 (9.6 kDa) is expressed as a His ${}_{6}$ –GB1 fusion protein containing a tobacco etch virus (TEV) cleavage site. Following expression of the protein in an *E. coli* BL21 (DE3) Δ EntD competent cell line (courtesy of Christian Chalut and Christophe Guilhot, CNRS, Toulouse, France) in M9 minimal media containing 1 g L ${}^{-1}$ ${}^{15}{\mathrm{NH}}_{4}\mathrm{Cl}$ and 2 g L ${}^{-1}$ ${}^{13}C$ glucose, the protein is lysed, purified through His affinity, cleaved with TEV protease to remove the GB1 tag, and purified by size-exclusion chromatography in phosphate buffer (20 mM sodium phosphate, pH 6.60 at 22 ${}^{\circ }$ C, 150 mM NaCl , 1 mM dithiothreitol (DTT), and 1 mM ethylenediaminetetraacetic acid (EDTA)). The protein was flash-frozen in liquid nitrogen and stored at -80 ${}^{\circ }$ C before use in the one-pot loading protocol.

#### 3.2.2 One-pot chemoenzymatic synthesis of Cys-loaded PCP1

**Figure A1:**
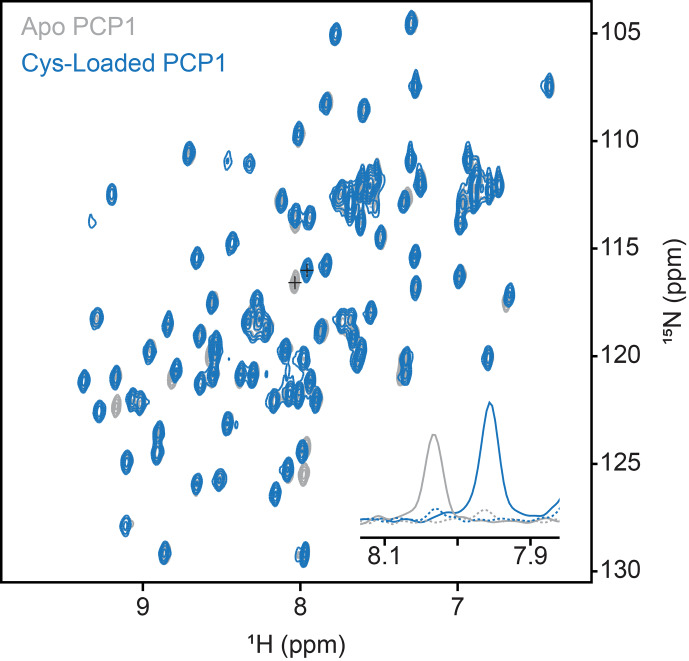
Conformation of loading of PCP1. Loading of PCP1 ( ${}^{15}N{/}^{13}C$ ) with the phosphopantetheine analogue was confirmed through 2-D HN-HSQC NMR. Upon loading of PCP1, the spectrum of apo PCP1 (gray) shows several characteristic peak shifts that can be used to monitor the chemoenzymatic loading of the prosthetic group (blue) detailed in Sect. 3.2. The inset shows 1-D slices of a PCP1 residue that reports on loading (peaks marked with crosses). Solid lines indicate slices at signal maxima and dashed lines indicate slices at the same position when signals are weak or non-existent.

**Figure A2:**
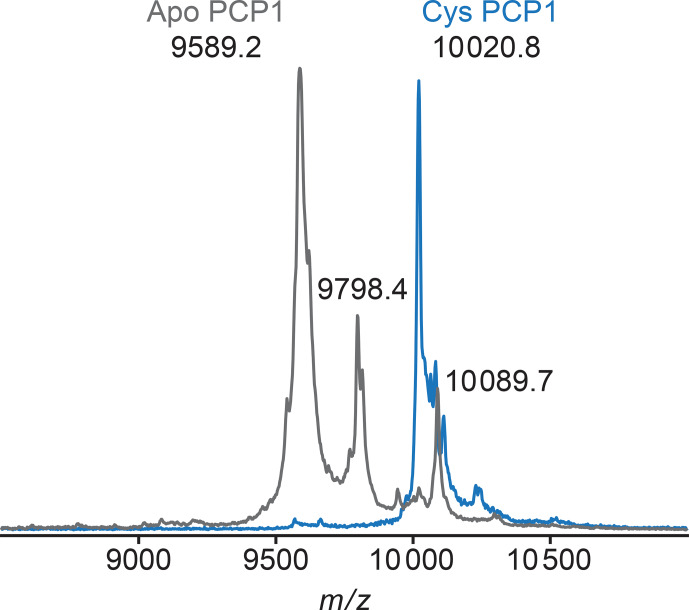
MALDI-TOF mass spectrometry is used to confirm loading of PCP1. To confirm loading of PCP1 with an orthogonal method, the mass-to-charge ratio of PCP1 approximates loading of the prosthetic group. Apo PCP1 shows a characteristic mass shift upon loading of the prosthetic. Here, an experimental mass change of 431.6 Da was observed. A theoretical mass change of 426.4 Da is expected upon loading of PCP1. Peaks at a m/z of 9798.4 and 10089.7 in apo are artifacts of MALDI sample preparation (e.g., matrix adduct with sinapinic acid). For comparison, the MALDI-TOF spectrum of Cys PCP1 has been scaled up by a factor of 3 as the sample was more dilute. All MALDI-TOF spectra were collected on a Voyager DE-STR (Applied Biosystems) spectrometer in linear mode using a mass range of 2500 to 25 000 Da. Sinapinic acid (10 mg mL ${}^{-1}$ in 50 % acetonitrile / 0.1 % trifluoroacetic acid, TFA) was applied as a sample matrix using a 10-fold dilution of protein sample in 0.1 % TFA. Spectra were calibrated against standards ranging from 5734.51 to 16952.30 Da (Protein Calibration Standard I, Bruker).

One-pot loading of apo PCP1 ( ${}^{15}N{/}^{13}C$ ) with the cysteine-linked pantetheine analogue (courtesy of David Meyers and Yousang Hwang, Johns Hopkins chemistry core facility) followed methods described by Worthington and Burkart (2006) and improved by Kittilä and Cryle (2018) with the following adaptations. Apo PCP1 was thawed and buffer exchanged into a one-pot reaction buffer: 100 mM Tris pH 7.55 at 22 ${}^{\circ }$ C, 10 mM ${\mathrm{MgCl}}_{2}$ , 100 mM NaCl , and 2.5 mM DTT. To a 10 mL reaction at 25 ${}^{\circ }$ C, apo PCP1 (118 µM ), Cys-NH-pantetheine analogue (509.4 µM ), adenosine triphosphate (ATP) (1 mM), pantothenate kinase (PanK) (2.5 µM ), phosphopantetheine adenylyltransferase (PPAT) (2.5 µM ), dephosphocoenzyme A kinase (DPCK) (2.5 µM ), and *Bacillus subtilis* Sfp phosphopantetheinyl transferase R4-4 mutant (Sfp R4-4) (5.0 µM ) were mixed together following sequential addition-incubation steps as in Kittilä and Cryle (2018). Preparation of PanK, PPAT, and DPCK was performed as described (Goodrich et al., 2017). Sfp R4-4 (courtesy of Jun Yin, Georgia State University) was prepared as described in Yin et al. (2006). The reaction incubated in a water bath at 25 ${}^{\circ }$ C for 2 h. Alkaline phosphatase (Calf Intestine, Quick CIP, New England BioLabs) was added to 1.0 U mL ${}^{-1}$ , and the reaction was monitored by NMR until loading completion was observed. Following this incubation period, 2-D ${}^{1}H{-}^{15}N$ heteronuclear single-quantum correlation (HN-HSQC) NMR and matrix-assisted laser desorption ionization time-of-flight (MALDI-TOF) mass spectra were used to determine loading efficiency (Figs. A1 and A2). Trace amounts of apo PCP1 are estimated to be less than 1 % (Fig. A2). The reaction mixture was then loaded onto a 5 mL HisTrap column (GE Healthcare) to remove PanK, PPAT, and Sfp R4-4, all of which have a His ${}_{6}$ affinity tag. The eluted PCP1 was concentrated and purified by size-exclusion chromatography on a Superdex 75 16/60 pg column (GE Healthcare) equilibrated with a one-pot reaction buffer. Upon confirmation of loading, purified Cys-loaded PCP1 (10 kDa with the prosthetic group) was concentrated and then buffer exchanged into an NMR buffer containing 20 mM sodium phosphate pH 6.59 at 22 ${}^{\circ }$ C, 150 mM NaCl, 1 mM EDTA, and 2 mM tris(2-carboxyethyl)phosphine (TCEP). All NMR experiments were performed on a 314 µM sample of PCP1 containing 10 % $D_{2}O$ and 200 µM sodium trimethylsilylpropanesulfonate (DSS). PCP1 concentration was quantified using UV–visible absorbance at 280 nm using an extinction coefficient of 6990 M ${}^{-1}$ cm ${}^{-1}$ .

### 3.3 Data acquisition

**Figure 3:**
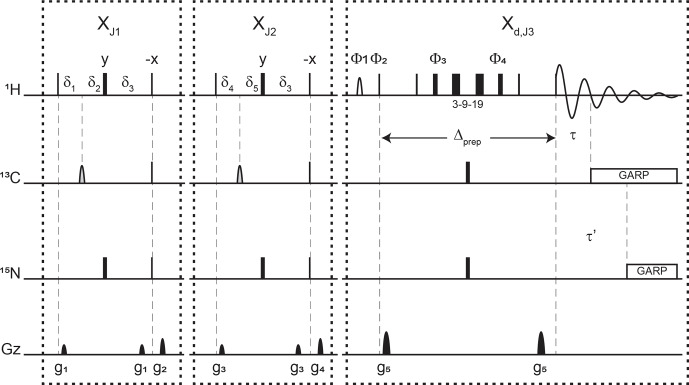
Pulse sequence for a 1-D double X half filter with delayed decoupling for suppression of ${}^{13}C$ (and/or ${}^{15}N$ ) labeled protein signals and preservation of unlabeled moieties signals. Dashes indicate different filtering blocks that can be used modularly to suppress scalar coupled spin systems. Here, X denotes isotopic half filters and subscripts J1, J2, and J3 indicate individually targeted scalar couplings. The subscript d indicates our delayed decoupling method. Thin and thick rectangles indicate 90 and 180 ${}^{\circ }$ hard pulses, respectively. Unless otherwise noted, the phase of these pulses is along the x axis. Open, half ellipses pulses on carbon are 180 ${}^{\circ }$ frequency-swept chirp inversion pulses (Böhlen and Bodenhausen, 1993), with durations of either 500 µs , when using sequential-tuned filters, or 1730 µs , when using the chemical shift coupling matched sweeping rate (Zwahlen et al., 1997). In Bruker, the shaped pulses were Crp60, 0.5, 20.1, and Crp60_xfilt.2, respectively. Water suppression is achieved through a WATERGATE element in the X ${}_{d,J3}$ filter employing a 3–9–19 suppression scheme (Piotto et al., 1992). A water flipped-back pulse (Grzesiek and Bax, 1993) (denoted with a curved open half ellipse) is applied on resonance with water (4.699 ppm) using a 1.5 ms 90 ${}^{\circ }$ sinc pulse. The delays in the X ${}_{J1}$ and X ${}_{J2}$ filter blocks are $\delta_{3}=1/|4\text{J}(\mathrm{NH})|\approx 2.78$ ms, $\delta_{1}=1/|4\text{J1}(\mathrm{CH})|$ , $\delta_{4}=1/|4\text{J2}(\mathrm{CH})|$ , $\delta_{2}=1/|4\text{J}(\mathrm{NH})|-1/|4\text{J1}(\mathrm{CH})|$ , and $\delta_{5}=1/|4\text{J}(\mathrm{NH})|-1/|4\text{J2}(\mathrm{CH})|$ . The delays τ and $\tau {}^{\prime }$ in the X ${}_{d,J3}$ delayed decoupling block can each be manually set to target residual ${}^{1}H{-}^{13}C$ and ${}^{1}H{-}^{15}N$ signals, respectively. Although we did not need this feature, residual ${}^{1}H{-}^{15}N$ signals can be suppressed using a shared delayed decoupling element that is included in our code. When suppression of surviving ${}^{1}H{-}^{15}N$ signals is not needed, both the 180 ${}^{\circ }$ pulse and decoupling sequence on the nitrogen channel can be switched off. The two delays τ and $\tau {}^{\prime }$ are arrayed to find the optimal level of suppression of targeted signals. Decoupling on carbon is performed by applying a globally optimized alternating-phase rectangular pulse (GARP) train (Shaka et al., 1985) on resonance with the targeted signals using a bandwidth of 2.083 kHz. Nitrogen decoupling is applied through a GARP sequence using a bandwidth of 1.042 kHz. The phase cycling in the delayed decoupling element is $\Phi_{1}=$ -x, x, $\Phi_{2}=$ x, -x, $\Phi_{3}=$ x, x, y, y, -x, -x, -y, -y, $\Phi_{4}=$ -x, -x, -y, -y, x, x, y, y, and $\Phi_{\mathrm{REC}}=$ x, -x, -x, x. Gradients are applied with lengths ( $\tau {\text{g}}_{i})$ and power ( ${\text{g}}_{i}$ ) of $\tau {\text{g}}_{1}=\tau {\text{g}}_{3}=\tau {\text{g}}_{5}=300$ µs (Bruker shape SMSQ10.32), $\tau {\text{g}}_{2}=\tau {\text{g}}_{4}=1$ ms (Bruker shape SMSQ10.100), ${\text{g}}_{1}=2.5$ G cm ${}^{-1}$ , ${\text{g}}_{2}=5.5$ G cm ${}^{-1}$ , ${\text{g}}_{3}=1.665$ G cm ${}^{-1}$ , ${\text{g}}_{4}=3.5$ G cm ${}^{-1}$ , and ${\text{g}}_{5}=30$ G cm ${}^{-1}$ . All gradients are followed with a 200 µs gradient recovery delay.

All NMR experiments were performed at 25 ${}^{\circ }$ C on a 600 MHz Bruker Avance III spectrometer equipped with a QCI cryoprobe using a 314 µM sample of PCP1 ( ${}^{15}N{/}^{13}C$ ) covalently modified with a non-hydrolyzable analogue of cysteine-linked phosphopantetheine (see Sect. 3.2). All 1-D and 2-D experiments were collected with 128 transients using 3072 points during detection. All 1-D spectra were zero-filled to 4096 points before Fourier transform and subsequently apodized using exponential multiplication with 7 Hz broadening to reduce truncation artifacts from buffer signals. In all experiments, water suppression was performed with a 3–9–19 WATERGATE element (Piotto et al., 1992) (see Fig. 3). Further details of water suppression and pulse/delay parameters can be found in Sect. 4.1. All 1-D spectra were processed in TopSpin 4.0.7 (Bruker BioSpin, 2019).

Implementation of the X ${}_{J1}$ X ${}_{J2}$ X ${}_{d,J3}$ combined filter into the 2-D ${}^{1}H{-}^{1}H$ TOCSY was done by incorporating our scheme into the WATERGATE sequence in a 2-D homonuclear X-filtered TOCSY pulse sequence (modifying the Bruker code dipsi2gpphwgxf) (Breeze, 2000; Iwahara et al., 2001; Ogura et al., 1996; Zwahlen et al., 1997). This pulse sequence includes t1 encoding, a TOCSY mixing time using a Decoupling in the Presence of Scalar Interactions 2 (DIPSI-2) sequence (Rucker and Shaka, 1989), followed by a sequential, double-tuned X filter and t2 encoding. A second version was coded to incorporate the X ${}_{d}$ block using an optimized τ of 431 µs . Both 2-D TOCSY spectra were collected with 1536 t1 and 128 t2 complex points using a spectral width of 16.0192 ppm in the detected dimension and 10.012 ppm in the indirect dimension. Quadrature detection in the indirect TOCSY dimension was achieved using the States method with time-proportional phase incrementation (States-TPPI) (Marion et al., 1989). The field strength of the DIPSI-2 TOCSY mixing sequence was 10.008 kHz and the mixing time was set to 40 ms. Both 2-D spectra were processed by zero-filling to 4096 points in the detected dimension and 1024 points in the indirect dimension. Before Fourier transform, spectra were apodized with a cosine-squared bell window function. The detected dimension was solvent-corrected using polynomial subtraction and extracted over the region from 9.0 to 6.5 ppm for focus on aromatic signals. All data were processed in NMRPipe (Delaglio et al., 1995) and referenced in both proton dimensions using the frequency of DSS.

## 4 Results and discussion

### 4.1 Description of pulse sequence elements

Figure 3 displays the pulse sequence elements used to assess the improvement provided by our method and, in the end, provide spectra of our unlabeled moiety free from signals of the labeled protein core. The novel element is labeled X ${}_{d,J3}$ , where d emphasizes that the filter ends during the detection, and J3 denotes the scalar coupling it targets. By analogy, we label each traditional X half filter as X ${}_{J1}$ and X ${}_{J2}$ , where J1 and J2 denote the couplings they target. When the Zwahlen method is used (Zwahlen et al., 1997), we label these periods X ${}_{Z}$ . In reference experiments, the X ${}_{d}$ block is replaced by a 3–9–19 water-suppression scheme, thus keeping all pulse sequences the same length for comparison. This consideration ensures that attenuations in signal intensities report exclusively on the efficiency of the filter and not on relaxation. The 3–9–19 scheme simply omits the inversion pulses on ${}^{13}C$ and ${}^{15}N$ shown in the X ${}_{d,J3}$ block, as well as the delayed composite pulse decoupling sequences.

Each of the X ${}_{J1}$ and X ${}_{J2}$ elements is an updated X half filter. When applied sequentially, they provide an update of the original sequential-tuned filter (Gemmecker et al., 1992) as described in Breeze (2000). Briefly, while single-quantum coherences in the isotopically labeled system evolve under scalar couplings, those in the unlabeled molecule will not. Thus, at the end of the delay δ3 , protons in labeled molecules feature antiphase coherences orthogonal to in-phase coherences of protons of unlabeled molecules. The desired in-phase coherences are then converted into longitudinal magnetization, while a pulsed-field gradient dephases the coherences of labeled molecules, now in multiple-quantum coherences. Variations in scalar couplings across the targeted molecule lead to imperfect signal suppressions, as occurs for protons coupled to ${}^{13}C$ in proteins, and several strategies have been developed to overcome this challenge (Breeze, 2000; Robertson et al., 2009). Two such strategies will form the basis for our comparisons.

When both blocks are applied sequentially with different values for J1 and J2, we obtain an updated version of the original sequential-tuned filter of Gemmecker et al. (1992). The principle is simple: multiple elements, each tuned to a target scalar coupling, are applied sequentially to cover a wider range of scalar couplings. With enough blocks, and with variations in tuned delays for each transient, remarkable editing can be achieved (Zangger et al., 2003) at the cost, however, of sensitivity losses due to relaxation during spin manipulations. In this study, to minimize such losses, only two blocks are considered. Our final implementation can be regarded as including a third block that is shared between the last preparation period and the detection period.

When using the chemical shift coupling matched sweeping rate in chirp pulses, we obtain a variant of the pulse sequence of Zwahlen et al. (1997). Here, the values of J1 and J2 are both set to a value of 147.4 Hz under our conditions, corresponding to τa=1.696 ms in the original paper. The main difference from the original paper is that the length of each block in our pulse sequence is maintained to 1/|2J(NH)| so as to permit comparisons with the sequential-tuned filter without interferences from relaxation losses. In either solution, time-shared filtering of amide protons is implemented (Burgering et al., 1993; Ikura and Bax, 1992).

The X ${}_{d}$ block implements our method into water-suppression schemes. Incorporation of water-suppression schemes in X half filters has already been described (Breeze, 2000; Sattler et al., 1999). Briefly, inversion pulses are applied on ${}^{13}C$ and ${}^{15}N$ concomitantly with the existing proton inversion, here in the form of a 3–9–19 sequence, to enable evolution under scalar couplings. In our strategy, composite pulse decoupling is then delayed until coherences have become antiphase during detection. The outcome of this strategy is to reduce the length of a filter preceding the detection period by sharing the evolution into antiphase coherences with the detection period, thus mitigating relaxation losses. For measurements in $D_{2}O$ , X half filters preceding the detection period can be implemented as X ${}_{d}$ blocks to reduce their lengths.

**Figure 4:**
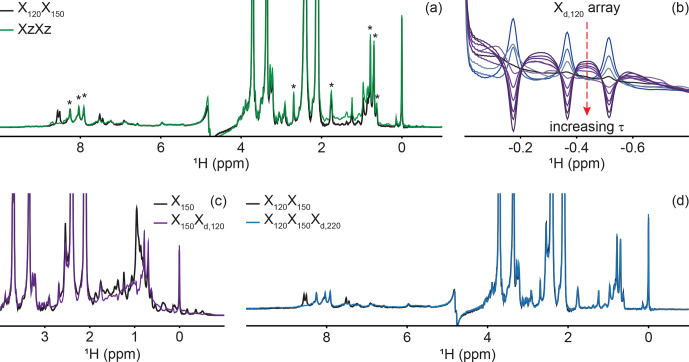
Delayed decoupling removes residual protein signals escaping traditional filters. **(a)** We compared suppression of aliphatic and aromatic signals from the core of PCP1 ( ${}^{15}N$ and ${}^{13}C$ labeled) by using a frequency-swept adiabatic pulse as referenced in Zwahlen et al. (1997) versus the level of suppression when employing a sequential double X half filter targeting 120 and 150 Hz in the aliphatic region. **(b)** Our delayed decoupling element can be arrayed to target specific scalar-coupled spins, here shown for PCP1 methyl peaks. τ ( µs ): 3 (blue), 1340, 1722, 2295, 2486, 3250, 3632, 4205, 4587, and 5542 (purple). $\Delta_{\mathrm{prep}}$ is set to 2.3939 ms. Optimal suppression is achieved for τ set to 2.295 ms corresponding to a total evolution time of 4.6889 ms (black). **(c)** Combining an X ${}_{150}$ block with an X ${}_{d,120}$ block (using the optimal value determined in panel **b**) suppresses methyl resonances but underperforms the X ${}_{150}$ X ${}_{120}$ scheme shown in panel **(a)** for scalar couplings departing from the targeted value. **(d)** Final X ${}_{120}$ X ${}_{150}$ X ${}_{d,220}$ filter with optimal suppression in both aromatic and aliphatic regions. τ was set to 329 µs , corresponding to a total evolution of 2.7229 ms. Peaks marked with asterisks in panel **(a)** indicate signals from the prosthetic group that are left untouched by the targeted filter elements. Other signals of unlabeled molecules belong to buffer components (EDTA and TCEP, with larger intensities) and DSS (at 0 ppm). Implementation of X ${}_{J1}$ , X ${}_{J1}$ X ${}_{J2}$ , X ${}_{Z}$ X ${}_{Z}$ , and X ${}_{J1}$ X ${}_{J2}$ X ${}_{d,J3}$ filters was performed as described in the pulse sequence (Fig. 3). The pulse sequences used to obtain all spectra that are compared have the same lengths, and the comparisons report exclusively on the efficiency of the filters.

### 4.2 Experimental implementation of delayed decoupling

A large range of ${}^{1}J_{\mathrm{CH}}$ scalar couplings hampers optimal suppression of coherences through traditional X-half-filter elements. We first set to compare the performance of established strategies for our system. We did not test low-pass filters, as Zwahlen at al. (1997) already demonstrated that their method outperforms it. Similarly, we did not implement improved sequential-tuned filters (Zangger et al., 2003), as we recognized that our method could provide a means to include the third filter needed for this improvement without an increase in pulse sequence length, and hence both methods should rather be combined than compared. Our two reference experiments are a double sequential-tuned half filter, X ${}_{150}$ X ${}_{120}$ , targeting 150 and 120 Hz scalar couplings, and the Zwahlen experiment X ${}_{Z}$ X ${}_{Z}$ (see Sect. 4.1 for departures from published sequences). Figure 4a reveals that each method is subject to orthogonal drawbacks. Globally, the Zwahlen method outperforms the sequential filter, as both aliphatic and aromatic regions are filtered. However, any chemical moiety with a deviation from the correlation between the scalar coupling and chemical shift used to optimize the sweep rate will display a residual signal, as observed most prominently in the aliphatic region. We note that we did not implement alternative means to exploit the correlation between scalar couplings and chemical shifts (Eichmüller et al., 2001; Kupče and Freeman, 1997; Stuart et al., 1999; Valentine et al., 2007), as outlier residues would still escape the filters, regardless of improvements in how the sweep is achieved. Figure 4a shows that using two sequential X filters tuned for 120 and 150 Hz outperforms the Zwahlen method in the aliphatic region. However, this advantage is offset by a near-complete lack of suppression for aromatic moieties (Fig. 4a, 6 to 9 ppm). Our observations reinforce previous comparisons (Zangger et al., 2003) but recording the references were necessary to identify signals escaping filters in our systems. In the remainder of this section, we will implement our X ${}_{d}$ block to a sequential-tuned filter to benefit from its superior suppression in the aliphatic region, whilst using X ${}_{d}$ to compensate for its deficiency in the aromatic region. Below, we implement X ${}_{d}$ gradually to combinations of X ${}_{J1}$ and X ${}_{J2}$ blocks to illustrate its performance and limitations, beginning with testing X ${}_{d}$ on its own.

Delayed decoupling is a tool to be combined with traditional X-filter elements to eliminate residual signals of labeled molecules. To test our X ${}_{d,J3}$ scheme and verify our theoretical predictions, we first incorporated it into a simple WATERGATE scheme (Piotto et al., 1992) and focused on isolated methyl resonances from PCP1 (Fig. 4b). This corresponds to a single X filter, including water suppression (Breeze, 2000; Sattler et al., 1999), taking place during both the preparation and detection periods. Here, we experimentally arrayed the delay for delayed decoupling, τ , to emulate our simulations (Fig. 2d) and observed near-complete suppression (black in Fig. 4b) when τ slightly exceeds the value calculated from the scalar coupling estimated from a non-decoupled spectrum (122 Hz), as discussed in Sect. 2. The differences between the line shapes of Figs. 2d and 4b reflect signal overlap and apodization. That all three resonances are suppressed at the same delay τ reflects how close their scalar couplings are. To illustrate the experimental limitations of our X ${}_{d}$ block, we paired it with a single X ${}_{150}$ block to make an X ${}_{150}$ X ${}_{d,120}$ sequence (Fig. 4c). Comparing the results with those of Fig. 4a for X ${}_{150}$ X ${}_{120}$ reports on the trade-off for including evolution into antiphase coherences during the detection period. In agreement with Sect. 2, X ${}_{150}$ X ${}_{d,120}$ achieves adequate suppression for the targeted scalar coupling (three isolated resonances also shown in Fig. 4b) but severely underperforms a conventional X ${}_{150}$ X ${}_{120}$ otherwise. Thus, although it serves its purpose to eliminate signals associated with specific scalar couplings, the method is not to be used as an alternative to existing methods.

**Figure 5:**
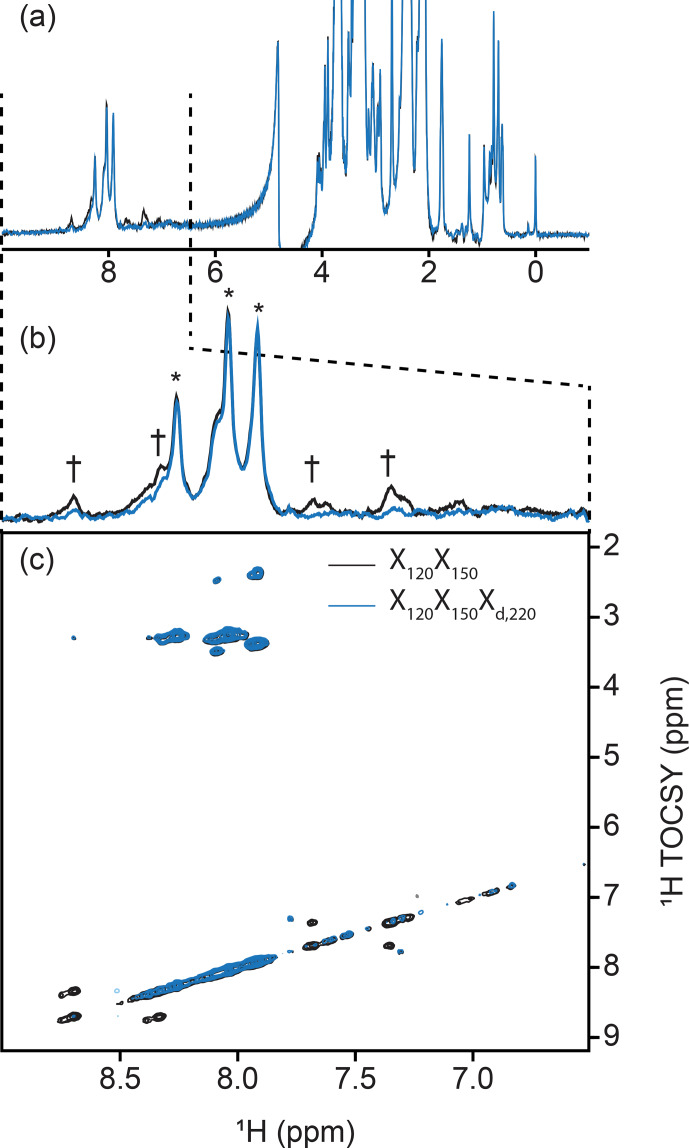
Implementation of delayed decoupling into 2-D homonuclear TOCSY. X ${}_{150}$ X ${}_{120}$ TOCSY was recorded with (blue) and without (black) a X ${}_{d,220}$ filter for enhanced suppression of aromatic signals. **(a)** 1-D trace of the detected dimension. The aliphatic region is identical in both spectra but X ${}_{d}$ improves the suppression of aromatic signals. **(b)** Zoom of the spectral region from 6.5 to 9.0 ppm emphasizing the reduction of aromatic signals. Daggers denote aromatic signals from PCP1 that are not suppressed in the TOCSY-X ${}_{120}$ X ${}_{150}$ experiment lacking X ${}_{d}$ . Asterisks denote signals from the prosthetic that are preserved. **(c)** Cross peaks between aromatic signals of PCP1 are largely suppressed in the 2-D TOCSY upon addition of our delayed decoupling technique with no loss in signal sensitivity for unlabeled signals.

Inclusion of delayed decoupling in the detected dimension of a sequential-tuned filter removes spurious signals with minimal costs in sensitivity and improves the quality of related multi-dimensional experiments. As revealed in Fig. 4a, the sequential X ${}_{150}$ X ${}_{120}$ sequence adequately suppresses signals in the aliphatic region but signals of aromatic protons escape filtering. Thus, we incorporated our X ${}_{d,J3}$ scheme to that sequence and coded an X ${}_{150}$ X ${}_{120}$ X ${}_{d,220}$ sequence. Effectively, this sequence can be regarded as a variation of the triple-tuned filters of Zangger et al. (2003), in which the last filter is shared between the water-suppression scheme and the detection period. Here, τ was arrayed, and optimal suppression was achieved for a value corresponding to 183.6 Hz. Figure 4d shows that X ${}_{d}$ could suppress surviving signals from the protein by up to 83 % in the aromatic region (calculated from intensities) and the sequence will permit unambiguous studies of the unlabeled tethered prosthetic group. As an immediate application, we show enhanced suppression of undesired PCP1 signals when X ${}_{d}$ is incorporated into the direct dimension of a sequential X-half-filtered 2-D TOCSY experiment (Fig. 5). To demonstrate the improvement, the 2-D TOCSY was first run using a conventional X ${}_{120}$ X ${}_{150}$ filter element (Fig. 5, black) and compared with a 2-D TOCSY using an X ${}_{120}$ X ${}_{150}$ X ${}_{d,220}$ element (Fig. 5, blue) that additionally targets residual signals in the aromatic region. A simple visual inspection reveals that spurious cross peaks between protein aromatic signals are efficiently suppressed upon addition of the delayed decoupling element, while cross peaks observed between the amide protons in the phosphopantetheine arm and aliphatic neighbors (around 3.5 ppm along the y axis) do not suffer from losses in sensitivity. Thus, in spite of its limitation and perhaps esoteric nature, we find delayed decoupling as a filter to be straightforward to implement and to immediately improve on existing filtering techniques aimed at studies of unlabeled moieties in presence of labeled partners.

## 5 Future directions

Delayed decoupling can readily be incorporated into existing water-suppression schemes and hence implemented into traditional multidimensional experiments, e.g., nuclear Overhauser effect spectroscopy (NOESY) or correlated spectroscopy (COSY), with detection of unlabeled moieties. Notably, X-filtering techniques are routinely employed with NOESY experiments to provide correlations between labeled and unlabeled moieties (van Ingen et al., 2002; Karimi-Nejad et al., 1999; Ogura et al., 1996; Otting and Wüthrich, 1990; Petros et al., 1992) and reveal binding sites or permit structure determinations of complexes. More generally, we anticipate this method will readily improve studies that focus on molecular responses of unlabeled moieties in presence of labeled partners. Ligand-binding studies should benefit from this advance, in particular for tight binding when alternatives based on translational and rotational diffusion will fail. We will make immediate use of this filtering methodology to monitor chemical modifications of carrier proteins, e.g., to monitor the progress of the reaction described in Fig. 1. Notably, although well established (Kittilä and Cryle, 2018; Worthington and Burkart, 2006), the method requires a series of active enzymes, and any defective component delays or abrogates the global reaction. With our improved scheme, we will be able to monitor steps of this reaction without interferences from residual protein signals. Further experiments using these improved filters will enable studies of interactions between the prosthetic arm and PCP1, in isolation and in the presence of its catalytic partner domains.

## 6 Conclusion

We have demonstrated that combining delayed decoupling with existing X half filters improves the suppression of labeled signals while preserving unlabeled signals in mixed samples. We have shown that the delayed decoupling technique can be easily shared between WATERGATE elements that are routinely used to study proteins by solution NMR and the detection period. Although only efficient over a narrow range of scalar couplings, and hence of little use as a stand-alone method, the method is complementary to existing filters. Specific scalar couplings that survive pre-existing X filters are optimally and easily suppressed by arraying a delay in the pulse sequence. We anticipate that our technique will facilitate studies of post-translational modifications or protein–small molecule interactions and will help monitor in situ chemical reactions targeting macromolecules.

## Data Availability

All NMR data including acquisition parameters, pulse sequences, and NMRPipe processing scripts, Matlab codes, and MALDI-TOF data are available for download from Zenodo at https://doi.org/10.5281/zenodo.4730836 (Marincin et al., 2021).
